# Carbazole Derivatives as Antiviral Agents: An Overview

**DOI:** 10.3390/molecules24101912

**Published:** 2019-05-17

**Authors:** Anna Caruso, Jessica Ceramella, Domenico Iacopetta, Carmela Saturnino, Maria Vittoria Mauro, Rosalinda Bruno, Stefano Aquaro, Maria Stefania Sinicropi

**Affiliations:** 1 Department of Pharmacy, Health & Nutritional Sciences, University of Calabria, 87036 Arcavacata di Rende, Italy; anna.caruso@unical.it (A.C.); r.bruno@unical.it (R.B.); stefano.aquaro@unical.it (S.A.); s.sinicropi@unical.it (M.S.S.); 2 Department of Science, University of Basilicata, 85100 Potenza, Italy; carmela.saturnino@unibas.it; 3 UOC Microbiologia e Virologia AO Annunziata, 87100 Cosenza, Italy; m.v.mauro@virgilio.it

**Keywords:** carbazole, tetrahydrocarbazole, antiviral agents

## Abstract

Viruses represent the most common cause of infectious diseases worldwide and those with rapid propagation and high infection rates cause human and animal pandemics. These fast-spreading diseases are generally treated with antiviral drugs but, often, drug resistance occurs because of the ability of the pathogens to mutate rapidly and become less susceptible to the treatments. Even though new antivirals have been approved, e.g., in HIV (human immunodeficiency virus) and HCV (hepatitis C virus) therapeutic areas, the need to dispose of new pharmaceutical tools for the management of infections that still have no treatment is of growing interest. In these areas, carbazole represents an important privileged scaffold in drug discovery. Many compounds with a carbazolic core have been developed and some of them have shown antiviral activity. This review provides an overview on some already known carbazole derivatives, pointing the attention on the running progresses in identifying new molecules with carbazolic structure, that have shown interesting and encouraging in vitro and in vivo properties. These drugs may be exploited as valid alternatives in antiviral therapy.

## 1. Introduction

Viruses are the cause of extremely widespread diseases, including the common cold, influenza, chickenpox, herpes, gastroenteritis (stomach flu), human immunodeficiency Virus (HIV/AIDS), and hepatitis. Viral diseases often bring severe and potentially life-threatening complications, indeed viral infections are likely responsible for more than 60% of the sicknesses affecting people living in developed countries [1].

Over recent decades, progress in several areas has been made in treating viral infections, for instance, regarding the drugs employed for the treatment of herpes simplex virus (HSV), hepatitis C virus (HCV), human immunodeficiency virus (HIV), and human cytomegalovirus (HCMV). However, in spite of these significant advances, different common viral infections still do not have effective and low-risk treatments [2].

For example, both the Dengue virus, which is the cause of 50–100 million cases of Dengue fever per year [3], and the oncogenic human papillomaviruses, which is the cause of cervical cancer [4], do not have an adequate and effective therapy. These observations clearly indicate that the search for new broad-spectrum antiviral agents with reduced side effects is a great scientific topic [5].

Several synthetic drugs are commercially available, but some natural compounds have demonstrated interesting antiviral activities; these molecules may be chemically modified in order to optimize the efficacy and/or selectivity and diminish the unwanted effects. Amongst these natural compounds, the alkaloid carbazole is one of the relevant structural motifs in drug discovery. Indeed, carbazoles have been largely investigated for their various biological properties (antibacterial, anti-inflammatory, psychotropic, anti-histamine, and antitumor) [6,7,8,9,10,11,12] and also antioxidant, antiviral, and neuroprotective activities [1,2,13,14,15,16,17,18,19].

Carbazole is a “privileged scaffold” because it serves as a ligand for many receptors and possesses the only property to bind reversibly to enzymes, providing a series of opportunities to study novel drugs that target one or more biological structures [19,20,21,22,23,24,25].

It must be highlighted that carbazoles gained an important position as worthy tools in different preclinical and clinical trials [26,27,28,29,30,31].

This review reports the most recent developments of carbazole derivatives in the relatively low-studied area of antiviral drug discovery, focusing on the activity of these compounds for the treatment of widespread infections produced by HIV, HCMV; hepatitis C virus (HCV), HSV, and human papilloma viruses (HPVs).

## 2. Human Immunodeficiency Virus (HIV)

The human immunodeficiency virus (HIV) is the etiologic agent of acquired immune deficiency syndrome (AIDS) and is a retrovirus (subclass of lentiviruses), in which the RNA genome allows higher genetic variability and rapid adaptability. Thirty-seven million people worldwide are infected by HIV, especially in Southern Africa, which has the highest percentage of infections [32].

Nowadays, HIV prevalence is increased because of antiretroviral therapy which allows a longer life, but, at the same time, the incidence of new infections has decreased from 3.3 million in 2002, to 2.3 million in 2012 [33]. In 2015, almost 15.8 million people were subjected to the antiretroviral therapy, amongst them ~41% of adults and ~32% of children [34].

The antiretroviral therapy (ART) is based on the co-administration of drugs that inhibit simultaneously multiple viral targets in order to strongly inhibit viral replication and minimize the onset of drug resistance. So far, six classes of antiretroviral drugs are available: nucleoside/nucleotide reverse transcriptase inhibitors (NRTIs), non-nucleoside reverse transcriptase inhibitors (NNRTIs), protease inhibitors (PIs), fusion inhibitors, CCR5 antagonists and integrase inhibitors (INIs). As already mentioned, a breakthrough has been made with the introduction of a therapy that combines different drugs to diminish the occurrence of resistance. Some studies reported that the use of a protease inhibitor together with two NRTIs, markedly reduced morbidity, and mortality [35,36,37].

However, therapies fail in almost the 8% of treatment-native and 33% of treatment-experienced patients [38]. The current drugs cannot eradicate the HIV-1 infection and their use is necessary lifelong; moreover some patients subjected to therapy during the past decades hold viral strains that are less susceptible to many available drugs [39].

Thus, the study of novel compounds with HIV activity is very attractive and it challenges research fields [40,41,42].

**Figure 1:**
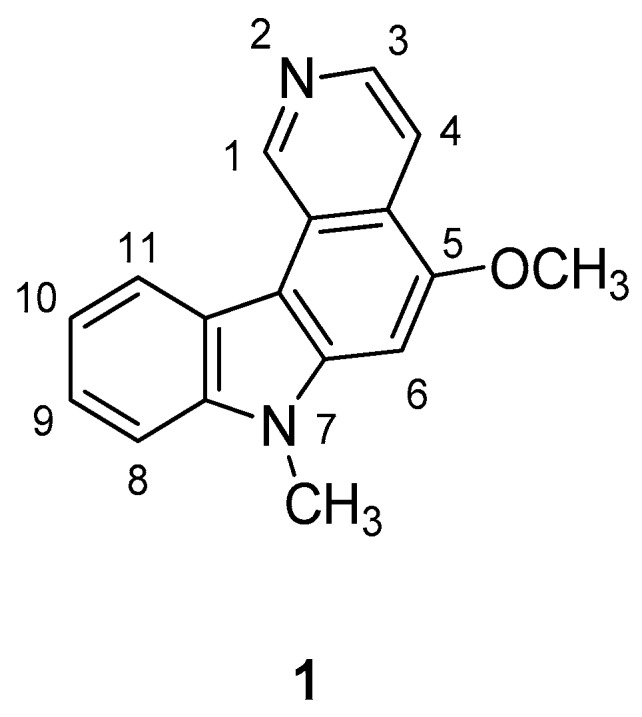
Structure of 5-methoxy-7-methyl-7*H*-pyrido[4,3-c]carbazole (**1**).

Hirata et al. in 1999 [43] have prepared a series of *N*-alkyl-pyrido[4,3-c]carbazoles starting from 2-hydroxy-9*H*-carbazole-3-carbaldehyde (mukonal), a chemical constituent present in Rutaceous plants. These compounds are structurally related to the antitumor alkaloid Ellipticine and to its synthetic analogs and they have been screened as antiviral agents. Several compounds showed interesting inhibition of HIV replication in H9 lymphocytes and the 5-methoxy-7-methyl-7*H*-pyrido[4,3-c]carbazole (**1**) showed an EC50 value of 0.0054 µg/mL and the highest therapeutic index (TI = 503) in the series, Figure 1.

The analogue compound with a chlorine at C-1 was less active (EC50 = 0.19 µg/mL), ten-fold less cytotoxic, and exhibited a lower TI (equal to 136.8). The hydroxylation at the C-10 lead to a decreased activity and TI (EC50 = 1.4 µg/mL, TI = 3.6) thus, the optimal combination of high activity and low toxicity occurred only with compound **1**, which has no substitution at C-1, being more active than Zidovudine (AZT) (EC50 = 0.012 µg/mL).

**Figure 2:**
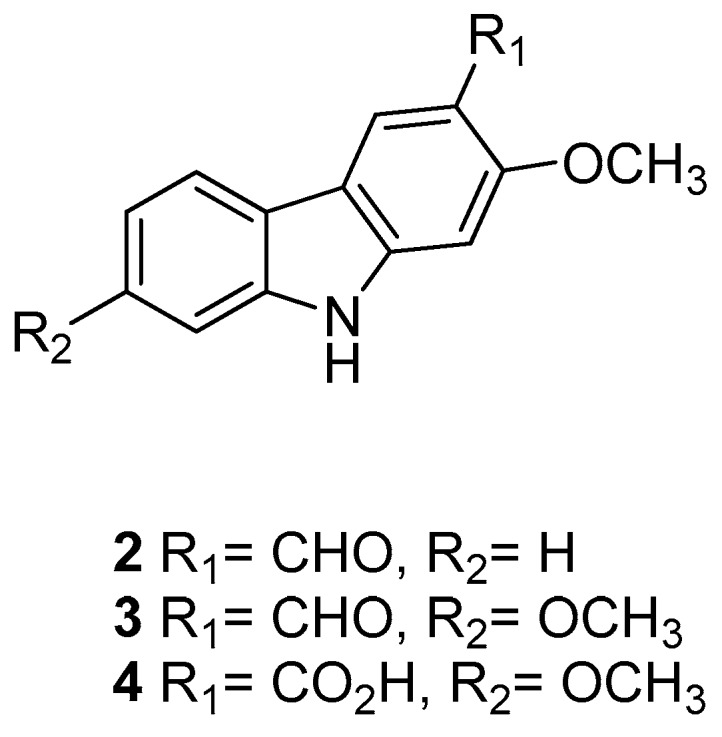
Structures of *O*-methylmukonal (**2**), 3-formyl-2,7-dimethoxycarbazole (**3**) and clauszoline J (**4**).

In 2005 Kongkathip et al. [14] reported the isolation, characterization, and anti-HIV-1 activity of three carbazoles, Figure 2: *O*-methylmukonal (**2**), 3-formyl-2,7-dimethoxycarbazole (**3**), and clauszoline J (**4**), from the *Clausena excavata* rhizomes and roots. Compounds **2**–**4** displayed a promising anti-HIV-1 activity with EC50 values of 12, 29.1, and 34.2 μM, respectively, and exhibited potential therapeutic index (PTI) values of 56.7, 8.0, and 1.6 respectively.

It was found that compounds **2**–**4** (200 μm) did not inhibit reverse transcriptase, exhibiting less than 30% of the HIV-1 reverse transcriptase activity. They probably target other stages of the HIV-1 replication cycle; however, the biological results support the use of the *C. excavata* extracts as remedies for the treatment of AIDS infections.

**Figure 3:**
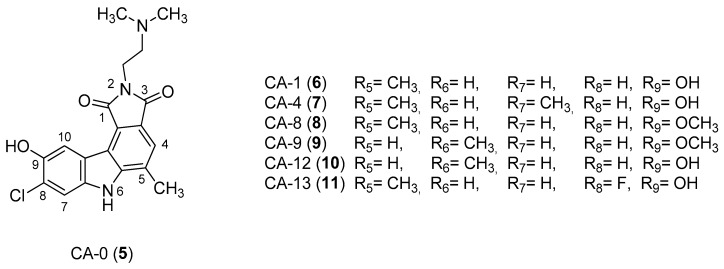
Structures of 8-chloro-2-[2-(dimethylamino)ethyl]-9-hydroxy-5-methylpyrrolo[3,4-c]carbazole-1,3(2*H*,6*H*)-dione (**5**) and derivatives **6**–**11**.

In 2005, Yan et al. [44] used a single replication infectivity assay with HeLa4.5/EGFP cells to investigate the antiviral activity of small molecular weight compounds with a carbazole structure, Figure 3. The IC50 values for CA-0 (**5**), CA-1 (**6**), CA-4 (**7**), CA-8 (**8**), CA-9 (**9**), CA-12 (**10**), and CA-13 (**11**) were 0.48, 0.92, 1.52, 0.79, 0.8, 0.69, and 0.51 μM, respectively. The IC50 values of the seven compounds were 5 to 10-fold lower than that of the strand transfer assay. However, the seven compounds were considerably toxic, suggesting different strategies for the development of carbazole-based inhibitors. Comparing the studied compounds, they recognized three important factors responsible for the inhibition: the most important factor was the incidence of a 2-dimethylaminoethyl group at position R2, then the incidence of a methyl group at the positions R5, R6, or R7, and the third was the nature of the substituent at position R9. In conclusion, the compound with a carbazole scaffold named 8-chloro-2-[2-(dimethylamino)ethyl]-9-hydroxy-5-methylpyrrolo[3,4-c]carbazole-1,3(2*H*,6*H*)-dione (**5**), was designed as lead compound to develop novel inhibitors.

**Figure 4:**
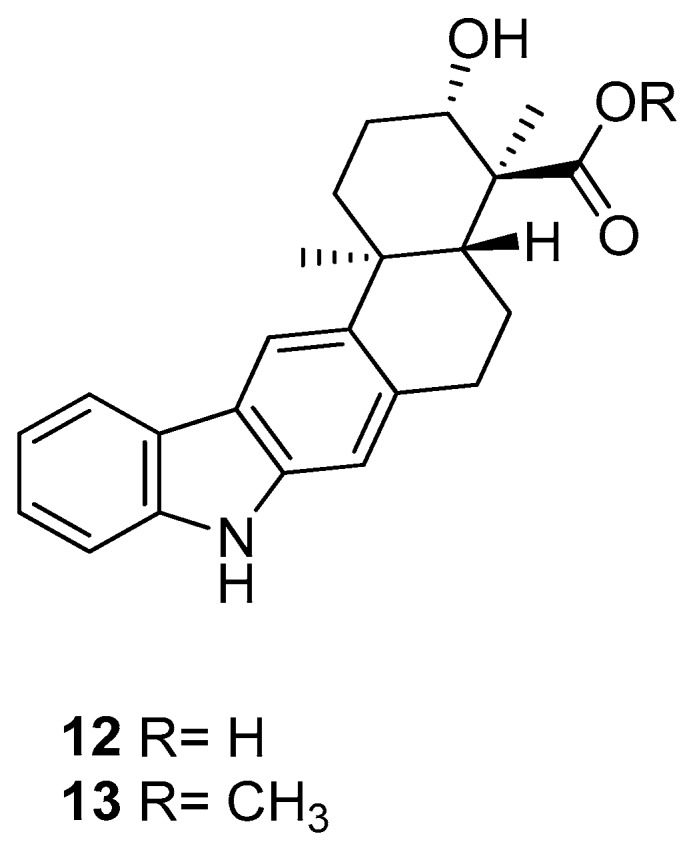
Structures of xiamycin (**12**), and its methyl ester (**13**).

In 2010, Ding et al. [45] obtained a pentacyclic indolocarbazole, Figure 4, named xiamycin (**12**), and its methyl ester (**13**), from Streptomyces sp. GT2002/1503, an endophyte from the mangrove plant Bruguiera gymnorrhiza. Xiamycin (**12**) is one of the first examples of indolosesquiterpenes isolated from prokaryotes; these sesquiterpenes are rare endophyte metabolites that might play an ecological role and, for this reason, the compound **12** exhibited a selective anti-HIV activity and may contribute to the antibiotic reservoir of the mangrove plants [1,46].

**Figure 5:**
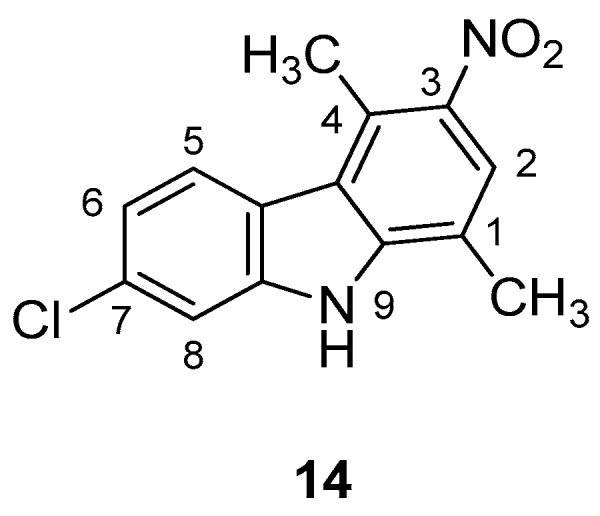
Structure of 7-chloro-1,4-dimethyl-3-nitro-9*H*-carbazole (**14**).

In 2018, Saturnino et al. [5] prepared a series of chloro-1,4-dimethyl-9*H*-carbazoles [47], which have been tested in CD4+, CXCR4+, and CCR5+ TZM-bl cells. Some compounds showed a moderate antiviral activity, although no significant differences were observed testing the compounds against CXCR4- or CCR5-using viruses. It seemed that the antiviral activity is due to the inhibition of different stages of HIV replication cycle. In details, the compounds bearing a chlorine at position 7 showed the greater activity against HIV, whereas the compounds with the chlorine at position 8 showed only a moderate antiviral activity. This indicates that the position of the halogen is vital for the antiviral activity. Moreover, the nitro compound 7-chloro-1,4-dimethyl-3-nitro-9*H*-carbazole (**14**), Figure 5, possessed the higher anti-HIV activity, thus in addition to the chlorine position on the carbazole scaffold the antiviral activity was improved by the presence of an electro-attractor group, in particular: NL4.3 X4 IC50 = 1.4 µM; Bal R5 IC50 = 5.3 µM; CC50 = 22.7 µM; S.I. = 4–16.

## 3. Human Cytomegalovirus (HCMV)

Human cytomegalovirus (HCMV) is a beta human herpes virus type 5 and is, probably, the cause of the most abundant human infections. In developed countries, the prevalence of HCMV has decreased thanks to improved sanitary conditions, but in low- and middle-income countries, almost all adults have contracted the infection during their youth. HCMV transmission can commonly happen, for instance, via saliva, sex, placental transfer, breastfeeding, blood transfusion, solid-organ transplantation, and in the human population HCMV serum levels range between 30% and 90% in developed countries, with a trend to increase with age [48,49].

In the U.S. and Northern Europe, the HCMV congenital infection is very frequent, affecting almost 30,000 infants, and is the cause of neurologic morbidity and many serious diseases as, for instance, the non-hereditary hearing loss and the neurodevelopmental disability [50,51,52] and, in fewer but significant cases, microcephaly, mental retardation, hepatosplenomegaly, and thrombocytopenic purpura [53].

Generally, in newborns infected by HCMV transmitted from the mother, the severity of the fetal infection strictly depends on the seropositivity or seronegativity for HCMV in mothers, the first one being the worse condition [54,55].

Nowadays, HCMV disease is faced using three approved compounds: ganciclovir (GCV), foscarnet (PFA), and cidofovir (HPMPC). However, as for other viral treatments, prolonged or repeated GCV therapy causes the onset of viral resistance and it is most frequent in highly immunosuppressed patients with great HCMV virus load. 

This phenomenon is relatively common and associated with the progression of the HCMV disease [56], thus the need to find other solutions to overcome the development of viral resistance have been reported [16,57].

**Figure 6:**
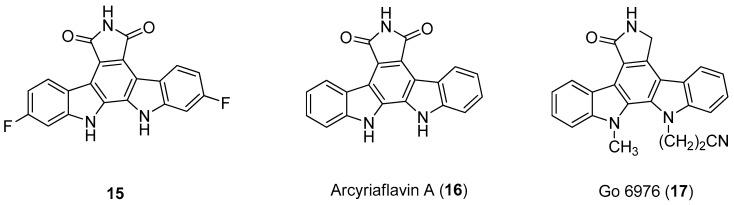
Structures of 12,13-dihydro-2,10-difluoro-5*H*-indolo[2,3-a]pyrrolo[3,4-c]carbazole-5,7-(6*H*)-dione (**15**), Arcyriaflavin A (**16**), and Go 6976 (**17**).

For this purpose, in 1999, Slater et al. [58] have identified some selective and powerful anti-HCMV compounds, Figure 6, particularly the 12,13-dihydro-2,10-difluoro-5*H*-indolo[2,3-a]pyrrolo[3,4-c]carbazole-5,7-(6*H*)-dione (**15**), which showed the best activity with an of IC50 = 40 nM and a therapeutic index >1450. These molecules were not good inhibitors of the protein kinase C βII and PKC, thus their activity to inhibit HCMV seems not to be related to the inhibition of viral protein phosphorylation. Additionally, they observed that Arcyriaflavin A (**16**) was a potent and selective inhibitor of HCMV replication and that Arcyriflavin A analogues were selective HCMV inhibitors. On the contrary, the antiviral activity of the analogue indolocarbazole lactam Go 6976 (**17**) was due to phosphorylation inhibition, in particular of PKC, which stops the viral replication in latently infected U1 cells [59]. 

The antiviral activity of indolocarbazoles resides in the inhibition of viral kinase UL97 or probably, in the inhibition of an induced or elevated host kinase [60,61]. Moreover, the co-treatment of Arcyriaflavin A (**16**) and GCV (Ganciclovir, 9-1,3-diidrossi-2-propossimetilguanina), brought to a potentiation of the antiviral effect. It is reasonable to say that the use of indolocarbazole inhibitors in combination with GCV in HCMV disease treatment may be a useful strategy.

**Figure 7:**
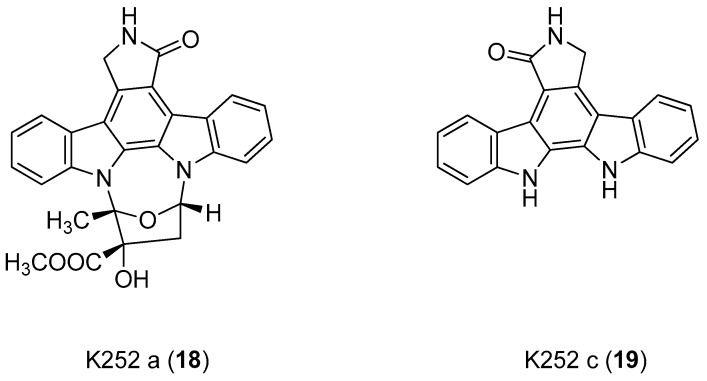
Structures of indolocarbazoles K252a (**18**) and K252c (**19**).

In 2000, Zimmermann et al. [16] analyzed a panel of protein kinase inhibitors (PKIs) and found that some indolocarbazoles (Go 6976 (**17**), Figure 6, K252a (**18**), and K252c (**19**), Figure 7) were good inhibitors of GCV-sensitive and -resistant HCMV strains, but no effect against the herpes simplex virus was observed. The antiviral activity was studied by focus reduction assays (0.009 µM < IC50 < 0.4 µM) and they observed that the indolocarbazoles treatment reduced the viral titer, after 5 days from infection, almost thrice. The effectiveness of these compounds strictly depended on the time post infection, indeed the higher activity was observed up to 24 h after the infection, with a significant reduction at 72 h. The biological target of indolocarbazoles was found to be the kinase pUL97, indeed they inhibit pUL97 autophosphorylation (0.0012 µM < IC50 < 0.013 µM) and pUL97-dependent ganciclovir phosphorylation (0.05 µM < IC50 < 0.26). Comparing indolocarbazoles with GCV, it is clear that they are highly active during the HCMV replication cycle and that the mechanism of action of the indolocarbazoles Go6976 (**17**) and K252c (**19**) is different from that of GCV. Most importantly, the authors have shown that the indolocarbazoles were effective against a GCV-resistant HCMV strain and showed low cytotoxicity. The inhibition of pUL97 autophosphorylation comes together with the ability to interact with the ATP binding site of Ser:Thr kinases, which shows the G–X–G–X–X–G motif in the domain II [62,63]. Considering that the interaction with this domain strongly blocks the enzymatic activity of pUL97, it is reasonable to suppose that an ATP binding site is essential for pUL97 functions. Summing up, these molecules possess a good antiviral activity and a low cytotoxicity, targeting the phosphorylation machinery of HCMV, particularly the pUL97 protein kinase.

## 4. Chronic Hepatitis C Virus (HCV)

Chronic hepatitis C virus (HCV) infection affects almost 71 million people worldwide and leads to severe morbidity and mortality as a result of liver diseases such as fibrosis, cirrhosis, and cancer [64], with about 3-4 million new cases of infection per year [65,66].

HCV infection prevalence is very variable, with the highest prevalence in Africa and the Middle-East and lower prevalence in the Americas, Australia, and Northern and Western Europe [67].

The most common route of HCV transmission is through drug abuse or, to lesser extent, by the use of contaminated instruments in surgery procedures [68].

HCV is one of the major public health issues for which no effective treatment is yet available, and the acute infection usually progresses to a chronic form that often leads to cirrhosis and hepatocellular carcinoma. Besides, HCV infection may produce several extra-hepatic diseases that affect kidneys, skin, salivary glands, eyes, thyroid, joints, and nervous and immune systems, with an incidence of 75% of patients, representing an important issue for health systems [69].

The common therapy for treating HCV infections includes ribavirin together with sofosbuvir or simeprevir, and, as well, pegylated α-interferon (PEG-IFN) [70], even though several side effects and drug resistances occur [71], thus there is a need to study and develop alternative anti-HCV drugs [72].

**Figure 8:**
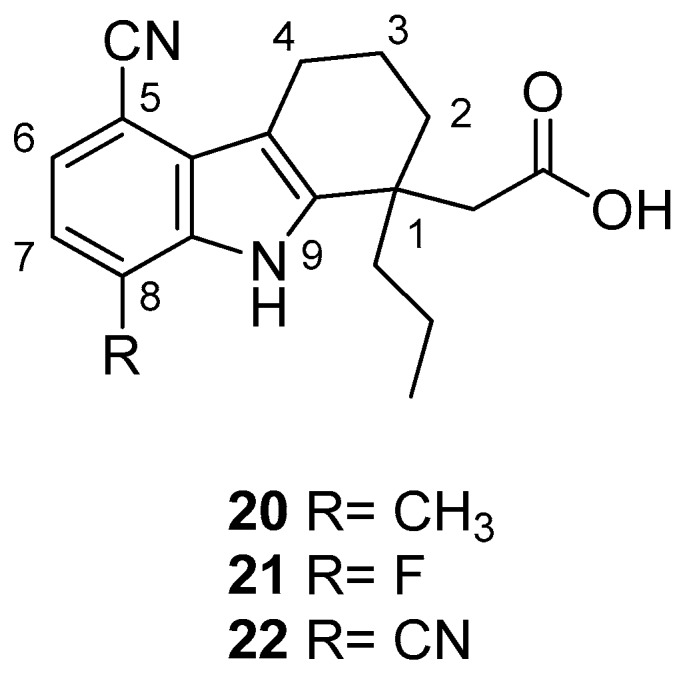
Structures of 2,3,4,9-tetrahydro-1*H*-carbazole derivatives **20**–**22**.

In 2006, Gopalsamy et al. [18] studied a class of RNA polymerase inhibitors containing a 2,3,4,9-tetrahydro-1*H*-carbazole scaffold, Figure 8. The optimization of the aromatic region showed preference for 5,8-disubstitution, favoring the *n*-propyl moiety in the C-1 position. From an earlier structure–activity relationships study on pyrano[3,4-b]indole [73], the C-1 *n*-propyl group was found to be the best in the aromatic region, thus they synthesized the carbazole analog **20** with appropriate aromatic substituents as those of the pyranoindole previously studied. The compound, obtained in racemic mixture, was less potent than the pyranoindole and the potency did not improve when the study was extended to other carbazoles bearing various aromatic substituents. Using a simple unsubstituted aromatic system, bearing the optimal substitution of n-propyl at the C-1, the inhibitory activity RNA polymerase was lost, indicating that aromatic substitutions are necessary. Even mono-chloro or di-fluoro analogs did not possess antiviral activity. However, a 5,8-disubstitution regained a substantial potency, with a preference for a methyl, a halogen, or a cyano group (for analogs **20**, **21,** and **22**, IC50 2.1 µM, 2.0 µM, and 2.3 µM respectively against HCV NS5B enzyme). In particular, compound **20** displayed inhibitory activities against the NS5B enzyme derived from HCV genotypes 1a, 1b, and 3a.

**Figure 9:**
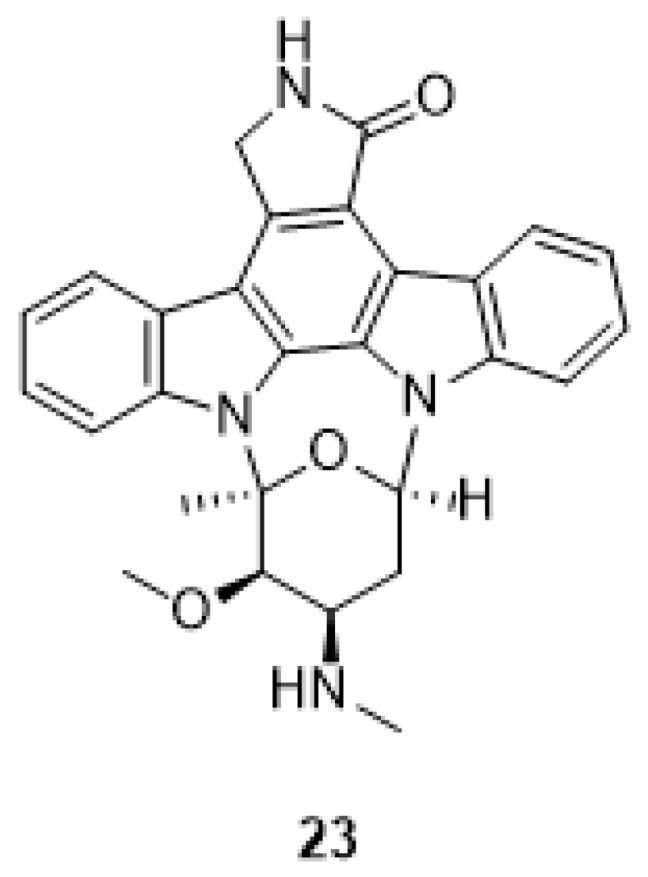
Structure of staurosporine (**23**).

In 2009, Murakami et al. [74] used the JFH1 viral culture system to screen some HCV inhibitors, finding out that indolocarbazoles block HCV replication independently from the PKC inhibition, which is one of the viral target of staurosporine (**23**), Figure 9, a broad-spectrum protein kinase inhibitor [75]. With the aim to develop different indolocarbazole inhibitors with higher potency and selectivity, the authors have tested panels of different indolocarbazole compounds but, again, the anti-HCV activity was not related to the inhibition of PKC. Other structurally related PKC inhibitors, such as the compound K252c (**19**) also inhibited HCV replication, however, the non-PKC-inhibitor arcyriaflavin A (**16**), [76], was also effective in reducing HCV RNA.

**Figure 10:**
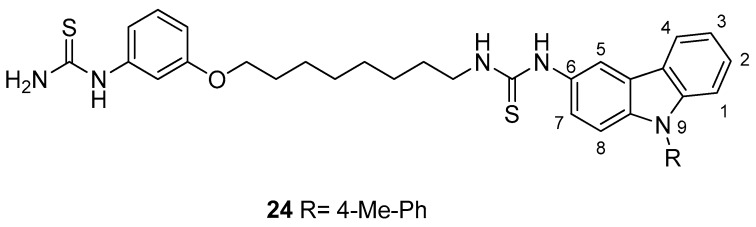
Structure of arylthiourea-carbazole derivative (**24**).

In 2009, Kang et al. [77] reported the synthesis and structure–activity relationships of some arylthiourea-carbazole derivatives with high inhibitory activity against HCV. Among the tested compounds, the carbazole derivative **24**, Figure 10, which possesses an eight-carbon linkage between the phenylic and carbazolic rings and a tolyl group at the *N*-9 of carbazole, exhibited a good anti-HCV activity, particularly on genotype 1b (EC50 = 0.031 µM), lower cytotoxicity (CC50 > 50 µM), and a high selectivity index (SI > 1612). The authors also investigated the effects of the substituents at *N*-9 of the carbazole moiety; the introduction of a methyl group at the *N*-9 position did not change the activity with respect to the *N*-H analogous derivative. On the contrary, the length of the alkyl substituent on the carbazole ring seemed to be important, because a progressive increased activity was found passing from methyl to hexyl substituent. It is reasonable to assume that this effect could be ascribed to the higher lipophilic properties of the compounds. The replacement of the alkyl group on the carbazole ring with a benzyl or phenylethyl one resulted, as well, in a potent activity against HCV. Interestingly, the addition of one (**24**) or two methyl groups on the *N*-phenyl ring produced an increased activity and no cytotoxic effects were observed. Finally, this series of carbazoles exhibited good anti-HCV activity and selectivity index.

## 5. Herpes Simplex Virus (HSV)

The two herpes simplex viruses, HSV-1 and HSV-2, are the cause of another widespread disease, which main manifestations are skin and mucous membrane sores or blisters [78]. Normally, HSV infections do not come along with serious complications but the wide viral diffusion is testified by the high number of seropositive persons [79].

The two HSV types differ by the antigenic envelope proteins, present a double-stranded DNA genome and are part of the alpha herpesviridae viruses’ subfamily. HSV lifelong infection may be asymptomatic when the immune system is working well but may evolve with recurrent lesions in the case of physical or mental stress. Generally, the type 1 is associated with oro-labial water blisters and is frequent during childhood, whereas the type 2 is almost entirely associated with genital herpes lesions, even though the latter may be caused by HSV-1, especially in Europe [80]. Genital HSV-2 infection is heavier than HSV-1 one and is associated with a more severe disease and symptomatic recurrences. HSV-2 seroprevalence shows a high variability across world nations, indeed in the USA HSV-2 infection rate is 22%, whereas it is lower in Europe (4 to 14%), with a higher presence in the north with respect to the south [81,82,83,84].

Nowadays, only three oral antiviral drugs are present: Aciclovir, that was the first drug for treating HSV, valaciclovir, an acyclovir prodrug, and famciclovir, which is the prodrug of the guanosine nucleoside analogue, penciclovir. These drugs share similar side effects, which include nausea, vomiting, headache, and diarrhea. Moreover, the onset of drug-resistance does not allow an efficient and prolonged treatment.

Another common human herpes virus is the Epstein-Barr virus (EBV), also named human herpes virus 4, a gamma-herpes virus. EBV infection is usually asymptomatic but may produce moderate to severe pathologies ranging from mononucleosis to several epithelial and lymphocytic cancers [85], for instance more than 200,000 cases of cancer each year are caused by EBV and almost 1.8% of deaths are due to EBV malignancies [86,87,88]. Moreover, in the past decade, it was estimated that EBV provoked almost the 10% of all gastric cancers [89].

**Figure 11:**
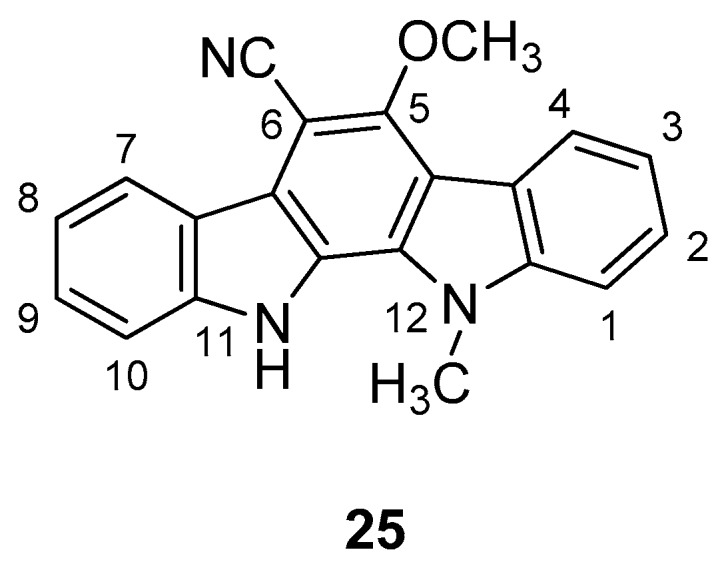
Structure of 6-cyano-5-methoxy-12-methylindolo [2,3-a]carbazole (**25**).

In 1990, Knübel et al. [90] have found that a plant extract of a blue-green alga *Nostoc sphaericum* (strain EX-5-1) showed a moderate antiviral activity against HSV-2. This extract contained some indolo[2,3-a]carbazoles, indeed the major component was the 6-cyano-5-methoxy-12-methylindolo [2,3-a]carbazole (**25**), Figure 11, and a second one, isolated and identified by NMR and HR mass spectral data, was the 6-cyano-5-methoxylindolo[2,3-a]carbazole, nor-*N*-methyl derivative of **25**. Compound **25** was obtained by ethanolic extract of the alga with successive normal and reversed-phase chromatography. The yield based on the dried alga weight was 0.22%. The antiviral activity and, as well, the cytotoxicity associated with the alga extract was due to the presence of compound **25**. Particularly, it was moderately active against HSV-2, as demonstrated by the reduction of about 95% at 1 µg/mL of the viral titer in infected mink lung cells. However, the virus was not totally eliminated at any concentration below the cytotoxic one (100 µg/mL), making it not useful as a valid lead candidate. The 6-cyano-5-methoxyindolo[2,3-a]carbazole showed similar biological profile.

**Figure 12:**
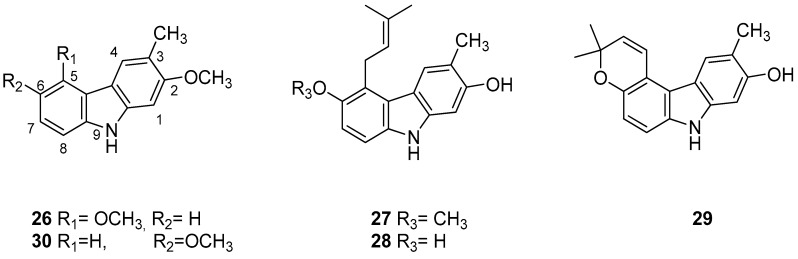
Structures of carbazole alkaloids from *Glycosmis arborea* (**26**–**30**).

In 2004, Ito et al. [91] studied the plant *Glycosmis arborea*, individuating three carbazole alkaloids, namely glybomines A (**26**), B (**27**), and C (**28**), and they isolated, from plant stems, some already-known monomeric alkaloids belonging to the carbazole, quinazoline, furoquinoline, quinolone, and acridone classes. Glybomine A (**26**) is the first example of a 2,5-oxygenated carbazole alkaloid from plants. The alkaloid’s antitumor activity was tested in a short-term in vitro assay of 12-*O*-tetradecanoylphorbol-13-acetate (TPA)-induced Epstein-Barr virus early antigen (EBV-EA) activation in Raji cells. All the tested alkaloids showed powerful dose-dependent inhibitory effects on EBV-EA induction by TPA (75.7–97.3% inhibition at 1 × 10^3^ mol ratio/TPA), together with low cytotoxic effects on Raji cells (even at 1x10^3^ mol ratio/TPA). Glybomine B (**27**) and glybomine C (**28**), possess a prenyl moiety at C-5 and showed a weaker inhibitory activity, even at 1 × 10 mol ratio/TPA (3.6–5.4%), but were more effective than glycoborinine (**29**) and glycozolidine (**30**). Thus, the presence of a prenyl group at the C-5 of the carbazole moiety is fundamental for the antitumor activity, Figure 12.

**Figure 13:**
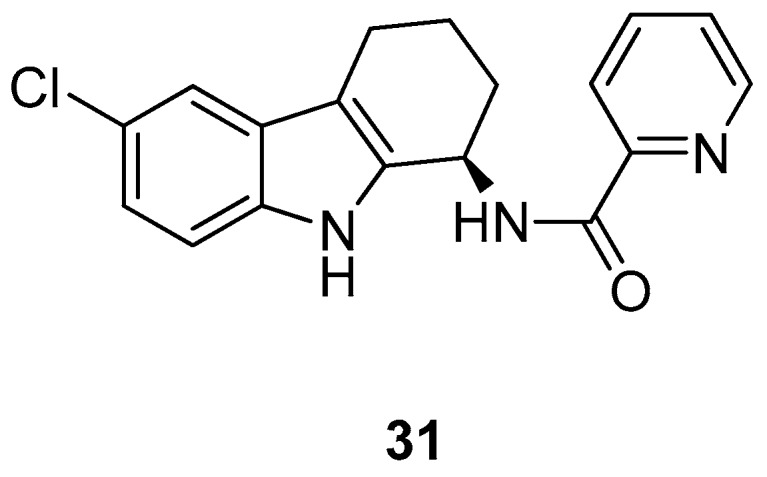
Structure of *N*-[(1*R*)-6-Chloro-2,3,4,9-tetrahydro-1*H*-carbazol-1-yl]-2-pyridinecarboxamide (GSK983, **31**).

In 2009, Harvey et al. [2] have described an antiviral compound, Figure 13, the *N*-[(1*R*)-6-chloro-2,3,4,9-tetrahydro-1*H*-carbazol-1-yl]-2-pyridinecarboxamide, named GSK983 (**31**), which showed an unique profile a broad-spectrum antiviral activity. In effect, GSK983 (**31**) inhibited the replication of several and different viruses in in vitro assays, with EC50 values of 5–20 nM, as the adenovirus Ad-5, the polyoma virus SV-40, the episomal maintenance of human papillomaviruses (HPV), and the Epstein-Barr virus (EBV). Anyway, the compound did not inhibit the HSV-1 and the HIV, moreover it was useless in the case of the lytic replication of EBV, at the concentrations below 1 µM. GSK983 (**31**) inhibited the growth of cell lines immortalized by HTLV-1, EBV, HPV, SV40, and Ad-5 (EC50 values ranging 10 to 40 nM), and was able to induce apoptosis or cycle cellular arrest. GSK983 (**31**) also inhibited cell lines immortalized by non-viral mechanisms but had little effect on primary cells. The pattern of inhibition, which includes viruses and immortalized cells, suggests a host cell protein as target, rather than a viral one and preliminary studies indicated that GSK983 (**31**) may induce a subset of interferon-stimulated genes.

## 6. Human Papilloma Virus (HPV)

Human papilloma viruses (HPV) are the cause of the most prevalent sexually transmitted infection and are the primary cause of the pathogenesis of different types of cancer [92,93,94].

At least one type of HPV is contracted by the majority of sexually active persons but the infection is often transient, asymptomatic, and is resolved spontaneously [95].

In the USA, women are subjected to the HPV infection twice as much as much as men, with a peak infection time after becoming sexually active [96] and virtually all cervical cancer cases result from genital infection with HPV. Cervical cancer affects almost 500,000 women each year, with an incidence of 80% in developing countries [97]. Given the elevated diffusion of HPV infection in the developed countries, the early detection and the treatment of the precancerous lesions represent a major issue in order to contrast the viral diffusion [98]. However, the continuous screening and early treatment programs are not enough to successfully control the increase of cervical cancer incidence [99,100], therefore the discovery of new drugs for HPV treatment of is also urgent.

**Figure 14:**
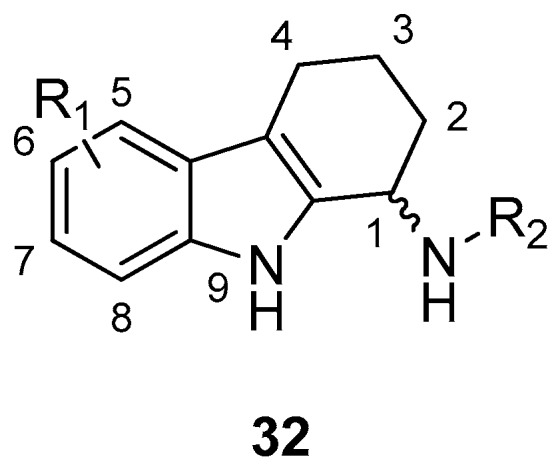
General structure of 1-aminotetrahydrocarbazoles (**32**).

**Figure 15:**
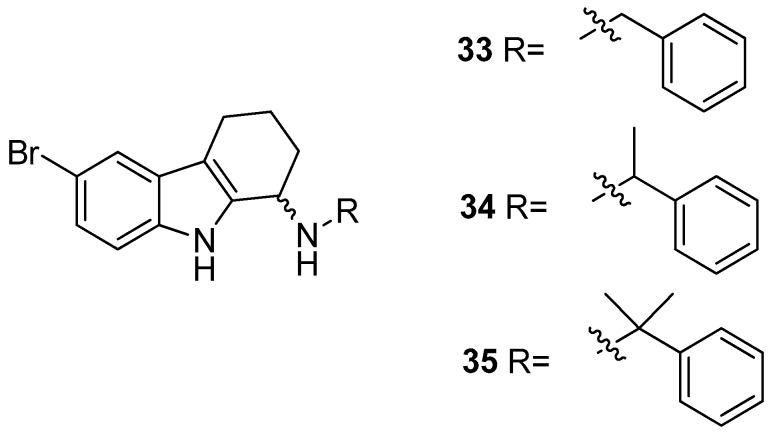
Structures of *N*-benzyl-6-bromotetrahydrocarbazoleamine (**33**), *N*-α-methylbenzyl-6-bromotetrahydrocarbazoleamine (**34**) and *N*-dimethylbenzyl-6-bromotetrahydrocarbazoleamine (**35**).

**Figure 16:**
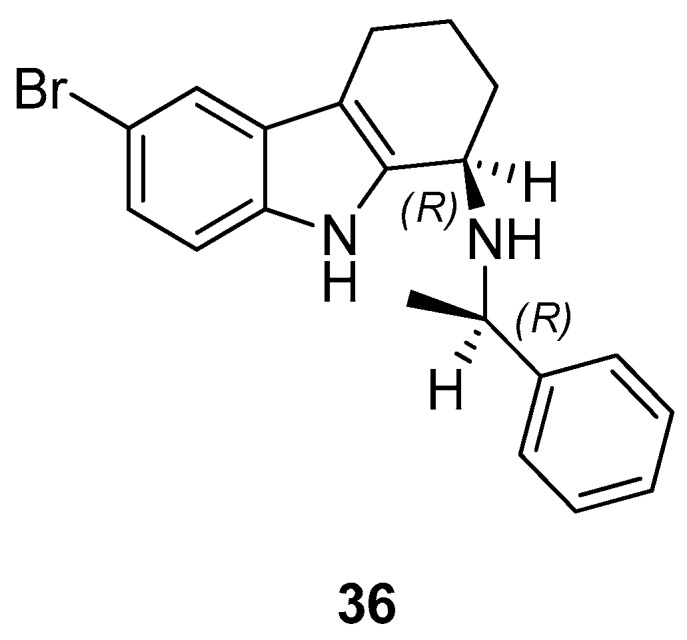
Structure of (1*R*)-6-bromo-*N*-[(1*R*)-1-phenylethyl]-2,3,4,9-tetrahydro-1*H*-carbazole-1-amine (**36**).

Gudmundsson et al. [17] described the synthesis and the structure–activity relationships of a series of substituted 1-aminotetrahydrocarbazoles (**32**) with high activity against human papillomaviruses, Figure 14. They focused on the activity of some tetrahydrocarbazoles bearing different substituents at C6, Figure 15. The presence of a lipophilic electron withdrawing substituent at C6 makes the compounds more active, being the bromo derivative (**33)** the most active (IC50 = 0.15 µM and CC50 = 18.8 µM). Moving the bromine at the C7 and C8 positions from the C6-position resulted in a two-fold reduction of the anti-HPV activity. Moreover, another role was played by the amine substituents of the 1-aminotetrahydrocarbazole, indeed the compounds with small alkylamine or polar amine substituents showed limited activity. The more lipophilic alkylaryl substituents, such as benzylamine (see **33**) or phenethylamine, showed a higher potency. Another improvement was made using the α-methylbenzylamine and indaneamine derivatives, indicating that α-substitution with alkylaryl groups was optimal. Notable is the very high anti-HPV activity of the α-methylbenzylamine (**34**) (IC50 = 0.03 µM; CC50 = 22.9 µM), in fact it is more powerful than the dimethylbenzylaminic derivative (**35**) (IC50 = 0.6 µM; CC50 > 40 µM). The promising activity of the α-methylbenzylaminic derivative (**34)** pushed the authors to synthesize its stereoisomers from the chiral (R)- and (S)- α-methylbenzylamines and the diastereomer (R,R) **36** (IC50 = 0.03 µM; CC50 = 9 µM) was selected for further studies, Figure 16. A cytotoxicity evaluation was made using human keratinocytes and Vero cells, and no toxic cellular effects were recorded (SI > 300 in human keratinocyte cells, SI > 500 in Vero cells) at the concentrations used for the antiviral activity. In vivo studies, using female rats, showed good pharmacokinetics (t_1/2_ 4.4 h; V_d_ 1.9 L/kg; F 47%; Cl 13.4 mL/min/kg), indicating viability of oral administration. Moreover, using the PanLab screening against a series of enzymes and receptors highlighted a low percentage of unwanted enzyme/receptor inhibition. Thus, the compound **36** was the most active against HPV and exhibited the lower cytotoxicity and the better pharmacokinetic profile, which makes it a good lead compound for further studies.

**Figure 17:**
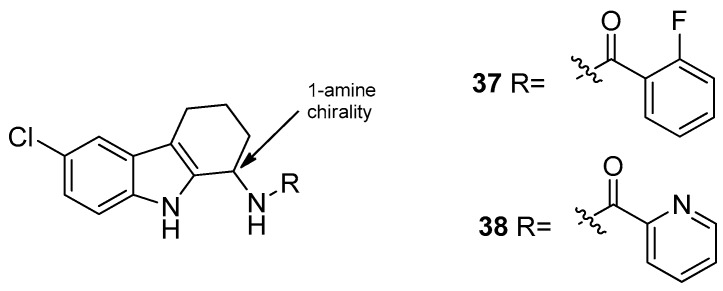
Structures of tetrahydrocarbazole amides **37**–**38**.

The same authors evaluated the improvement of the 1-aminotetrahydrocarbazole scaffold, showing that the presence of a lipophilic halogen (Cl or Br) at C6 and the α-methylbenzylamine substitution with (R)-stereochemistry at the 1-amine allowed a better anti-HPV activity (**36**). They also wondered whether the change of the basicity of the 1-amine would affect the anti-HPV activity. Therefore, they synthesized a series of tetrahydrocarbazole amides that exhibited, as well, a high activity against the human papillomaviruses. The 6-bromosubstituted tetrahydrocarbazole compounds showed the best activity, thus they synthesized the corresponding 6-chloro analogues with the aim to reduce the molecular weight and improve the pharmacokinetics. The 6-chloro compounds, Figure 17, mostly the (R)-enantiomers of the 2-fluorosubstituted benzamide (**37**) (IC50 = 0.008 µM; CC50 = 24 µM) and the 2-pyridinylamide (**38**) (IC50 = 0.005 µM; CC50 = 16 µM) demonstrated, again, a very impressive anti-HPV activity. Then, amongst the obtained compounds, the *N*-[(1*R*)-6-chloro-2,3,4,9-tetrahydro-1*H*-carbazol-1-yl]-2-pyridinecarboxamide (**38**) demonstrated the best profile and was employed for further evaluations [101].

## 7. Conclusions

The carbazole scaffold, commonly present in many biologically active pharmaceuticals and agrochemicals, is one of the most abundant heterocycles in nature. Several carbazole derivatives were individuated and in recent years an increase in interest in their use as bioactive molecules against different kinds of diseases has been recorded.

This review reports the current status and the recent developments of the most important carbazole derivatives in the antiviral drug discovery field and gives a comprehensive overview on carbazole antiviral agents employed in clinical trials, as well as currently evaluated in experimental studies (Table 1). Moreover, it summarizes the most important and recent results on the development of molecules with a carbazolic core that could represent the lead compounds for the development of new important antiviral drugs.

## Figures and Tables

**Table 1:** Carbazole derivatives as antiviral agents.

**Human Immunodeficiency Virus (HIV)**	
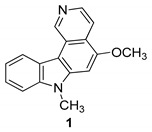	Hirata et al. 1999 [43]
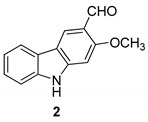	Kongkathip et al. 2005 [14]
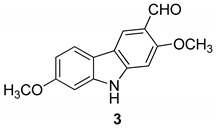	Kongkathip et al. 2005 [14]
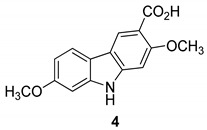	Kongkathip et al. 2005 [14]
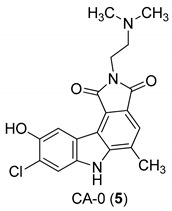	Yan et al. 2005 [44]
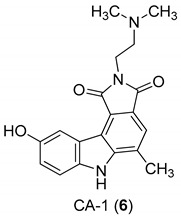	Yan et al. 2005 [44]
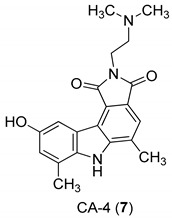	Yan et al. 2005 [44]
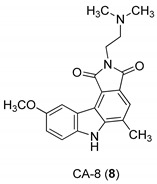	Yan et al. 2005 [44]
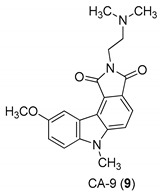	Yan et al. 2005 [44]
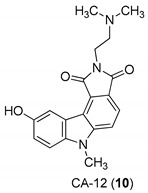	Yan et al. 2005 [44]
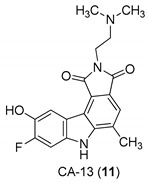	Yan et al. 2005 [44]
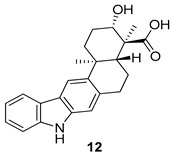	Ding et al. 2010 [45] Xu et al. 2012 [46]
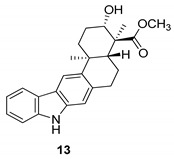	Ding et al. 2010 [45]
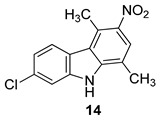	Saturnino et al. 2018 [5]
**Human Cytomegalovirus (HCMV)**	
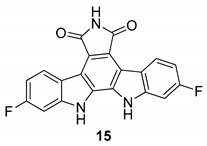	Slater et al. 1999 [58]
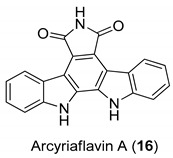	Slater et al. 1999 [58] Murakami et al. 2009 [74] Zhu et al. 2003 [76]
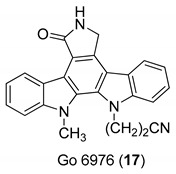	Zimmermann et al. 2000 [16] Slater et al. 1999 [58] Patzold et al. 1993 [59]
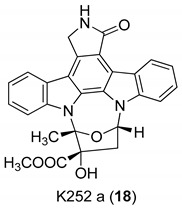	Zimmermann et al. 2000 [16]
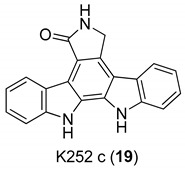	Zimmermann et al. 2000 [16]
**Chronic Hepatitis C Virus (HCV)**	
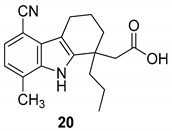	Gopalsamy et al. 2006 [18]
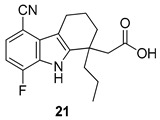	Gopalsamy et al. 2006 [18]
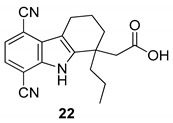	Gopalsamy et al. 2006 [18]
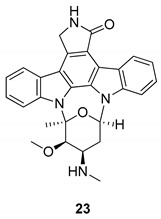	Murakami et al. 2009 [74] Wang et al. 2008 [75]
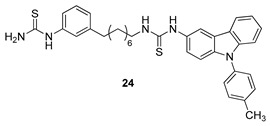	Kang et al. 2009 [77]
**Herpes Simplex Virus (HSV)**	
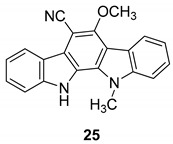	Knübel et al. 1990 [89]
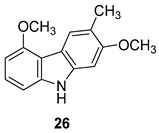	Ito et al. 2004 [90]
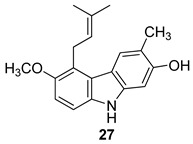	Ito et al. 2004 [90]
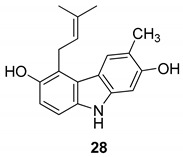	Ito et al. 2004 [90]
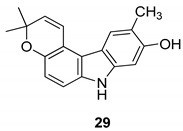	Ito et al. 2004 [90]
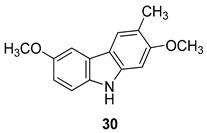	Ito et al. 2004 [90]
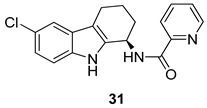	Harvey et al. 2009 [2]
**Human Papilloma Virus**	
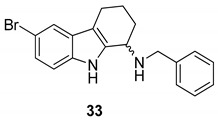	Gudmundsson et al. 2009 [17]
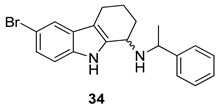	Gudmundsson et al. 2009 [17]
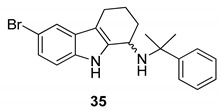	Gudmundsson et al. 2009 [17]
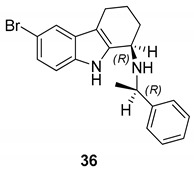	Gudmundsson et al. 2009 [17]
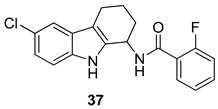	Gudmundsson et al. 2009 [100]
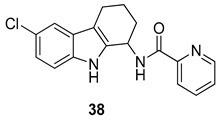	Gudmundsson et al. 2009 [100]
